# The predictive value of the Systemic Inflammatory Response Index in evaluating the severity of influenza A-induced viral pneumonia among elderly patients: a retrospective analysis

**DOI:** 10.3389/fmed.2026.1807561

**Published:** 2026-04-29

**Authors:** Piaopiao Wu, Jingping Cheng, Jiadun Wang

**Affiliations:** Department of Geriatrics, CR & WISCO General Hospital, Wuhan, China

**Keywords:** biomarker, elderly patients, influenza A virus, Systemic Inflammatory Response Index, viral pneumonia

## Abstract

**Background:**

Influenza A virus (IAV) pneumonia poses a significant threat to the elderly, often leading to severe outcomes due to age-related immunological decline. Early and accurate assessments of disease severity is critical for improving clinical management and prognosis. The Systemic Inflammatory Response Index (SIRI), which integrates neutrophil, monocyte, and lymphocyte counts, may serve as a novel biomarker to reflect the systemic inflammatory state and predict disease progression in older adults.

**Methods:**

A retrospective analysis was conducted involving 160 patients aged 65 years or older who were diagnosed with IAV pneumonia at China Resources Wuhan Iron and Steel General Hospital between December 2024 and March 2025. Patients were categorized into favorable (CURB-65 [confusion, urea, respiratory rate, blood pressure, age ≥ 65 years] score <3, *n* = 71) and unfavorable (CURB-65 score ≥3, *n* = 89) prognosis groups. Demographic, clinical, and laboratory data were collected. The SIRI was calculated as (neutrophils × monocytes)/lymphocytes. Statistical analyses were performed using *t*-tests, chi-squared tests, logistic regression, and receiver operating characteristic (ROC) curve analysis.

**Results:**

SIRI levels were significantly higher in the unfavorable prognosis group than in the favorable prognosis group (*p* < 0.05). Additionally, a positive correlation was found between SIRI levels and CURB-65 scores (r = 0.433, *p* < 0.001). A multivariate logistic regression analysis confirmed that the SIRI is an independent predictor of disease severity after adjusting for gender, age, and smoking history (*p* < 0.05). The ROC analysis demonstrated that the SIRI had an area under the curve of 0.806 for predicting severe disease, outperforming traditional inflammatory markers such as C-reactive protein, interleukin-6, and erythrocyte sedimentation rate.

**Conclusion:**

The SIRI is a reliable and objective biomarker for assessing disease severity and predicting prognosis in elderly patients with IAV pneumonia. Its use may facilitate early identification of high-risk individuals, guide timely clinical intervention, and ultimately improve patient outcomes.

## 1 Background

Pneumonia is a widespread respiratory disease that primarily affects the Elderly and young children with underdeveloped or weakened immune systems, making them the most vulnerable groups. Influenza A virus (IAV) is a common pathogen that causes community-acquired pneumonia and seasonal outbreaks every year (1). Its high transmissibility and transmission through the respiratory tract allow IAV to persist among the human population worldwide, resulting in significant morbidity and mortality. Each year, over 10,000 people die as a result of this virus (2). Influenza A virus pneumonia is an acute respiratory infectious disease, mostly caused by IAV infection, and it is characterized by alveolar and interstitial lung inflammation, making it a significant health hazard for the elderly (3).

Influenza-infected elderly patients face increased risks due to immunosenescence, the presence of underlying comorbidities (chronic cardiorespiratory diseases and diabetes), and a greater likelihood of multidrug-resistant infections. These factors contribute to their vulnerability to severe pneumonia, which can manifest as exacerbations with respiratory failure, septic shock, or multiple organ dysfunction syndrome (4). Influenza A virus is identified as the primary causative agent of severe pneumonia in this population, influenced by several known factors (5). According to the most recent estimates of the World Health Organization (WHO), the annual incidence of influenza ranges from 3 to 5 million cases worldwide, with 60% occurring in individuals over 65 years of age. The case fatality rate of the disease ranges between 10 and 30% (6). With the increase in the global aging population, the incidence of influenza A virus pneumonia in the elderly is expected to rise, posing a significant medical and family burden (7). Furthermore, due to the rapid progression of influenza A virus pneumonia, early diagnosis of serious disease outcomes plays an important role in improving the mortality associated with this condition. Early and quantitative evaluation of disease severity and prognosis in older adults with influenza A pneumonia is significant for clinical treatment, assessing treatment effectiveness, and promoting recovery.

The Systemic Inflammatory Response Index (SIRI) incorporates neutrophil, monocyte, and lymphocyte counts. It is an all-encompassing index that, according to theory, can provide a better overview of systemic inflammation by dynamically quantifying the relative abundance of major immune cell subsets in real time. The SIRI can be calculated by multiplying the neutrophil count by the monocyte count and then dividing by the lymphocyte count. There is increasing evidence that the SIRI is a powerful prognostic biomarker in multiple clinical scenarios, such as cardiovascular diseases, cancers, and sepsis (8). However, its applicability in respiratory viral infections, particularly in elderly patients with influenza A pneumonia, remains to be determined. As the SIRI represents the sum of contributions of the immune cell groups that participate in the host immune response upon viral infection, it may provide a more comprehensive expression of the “inflammation-immune” network imbalance in the elderly than each biomarker. Thus, it could serve as an early predictive factor for severe disease. Additionally, due to immunosenescence, the changes in immune cell composition associated with aging may be further exaggerated, which could further increase the sensitivity and clinical value of the SIRI in the older population.

Thus, in this study, we aim to explore the correlation between the new immune-inflammatory index SIRI and illness severity in older hospitalized patients suffering from influenza A virus-associated pneumonia. We also seek to clarify this correlation to guide clinicians in using more accurate early warning indicators for early detection, timely treatment, and risk prevention. Ultimately, the aim is to decrease the prevalence of severe disease and death in the elderly population, which is particularly vulnerable, and to decrease the burden on public health systems.

## 2 Materials and methods

### 2.1 Diagnostic criteria

Diagnostic criteria for community-acquired pneumonia: The diagnostic criteria were established in accordance with the “Guidelines for the Diagnosis and Treatment of Community-Acquired Pneumonia in Chinese Adults (2016 Edition)” (9): (1) onset of symptoms in the community setting; (2) a new cough, production of sputum, or worsening of existing respiratory symptoms, possibly accompanied by purulent sputum, chest pain, shortness of breath, or coughing up blood; (3) presence of fever; (4) clinical indicators of lung consolidation and/or the presence of moist rales upon auscultation; (5) peripheral blood white cell count greater than 10 × 10^9^/L or less than 4 × 10^9^/L, with or without a left shift in neutrophils; and (6) Chest imaging results show new patchy infiltrates, lobar or segmental consolidation, ground-glass opacities, or interstitial changes, possibly accompanied by pleural effusion. A diagnosis of community-acquired pneumonia can be confirmed if criteria (1) and (6) are fulfilled, along with at least one of criteria (2) to (5), and conditions such as pulmonary tuberculosis, lung cancer, non-infectious interstitial lung disease, pulmonary edema, atelectasis, pulmonary embolism, eosinophilic lung infiltration, and pulmonary vasculitis have been excluded.

Diagnostic criteria for influenza A virus-induced viral pneumonia include: (1) fulfillment of the diagnostic criteria for community-acquired pneumonia and (2) a positive outcome from either a rapid antigen detection test or a real-time fluorescence RT-PCR nucleic acid test for the influenza A virus with concurrent exclusion of other known viral causes of pneumonia, including influenza B virus, parainfluenza virus, adenovirus, respiratory syncytial virus, coronavirus, and human metapneumovirus.

Inclusion criteria included: (1) individuals between the ages of 65 and 95 who meet the diagnostic standards for community-acquired pneumonia (CAP) as outlined in the “Guidelines for the Diagnosis and Treatment of Community-Acquired Pneumonia” in Chinese Adults (2016 Edition), (2) patients diagnosed with influenza A according to established clinical criteria (10), and (3) patients willing and capable of completing all required ancillary examinations. Exclusion criteria included: (1) patients with concomitant malignant tumors, severe cardiovascular, hepatic, or renal dysfunction, or underlying conditions affecting the hematopoietic, immune, or endocrine systems; (2) patients with a history of lower extremity venous disease, long-term bed rest, recent surgery, trauma, radiotherapy, chemotherapy, or current use of anticoagulant or thrombolytic agents; (3) patients unable or unwilling to undergo blood sampling; and (4) patients with concurrent infections caused by other pathogens (all patients underwent respiratory multiplex PCR testing upon admission to exclude coexisting viral or bacterial etiologies).

### 2.2 Research methods

At 2 h after admission, blood samples of 4 mL were taken from patients in two group for examination including the whole-blood cell count, which includes white cells count (WBC), neutrophils count (NEU) and percentage, eosinophils count and percentage and monocyte cell count (MONO) and percentage, and lymphocyte count (LYM) and percentage, platelet count and hemoglobin level (Hb), C-reactive protein (CRP), procalcitonin, interleukin-6 (IL-6), erythrocyte sedimentation rate (ESR), prealbumin (PRE_Alb), and albumin (alb) were analyzed through an automated hematology analyzer. Total protein (TP), liver and kidney function indexes, direct bilirubin (DBIL), total bilirubin (TBIL), alanine aminotransferase (ALT), aspartate aminotransferase (AST), BUN, creatinine (Cr), uric acid (UA), myocardial enzyme spectrum creatine kinase, lactate dehydrogenase (LDH), and BNP were also detected. In addition, we obtained 2 mL of blood samples from the arterial of each patient for determining the PaO₂ and PaCO₂ partial pressures by a blood gas analyzer.

Calculation of new immune-inflammatory indicators: SIRI was calculated based on routine blood test and general laboratory data. Formula: SIRI = (neutrophil number times monocyte number)/lymphocyte number, the unit is expressed as ×10^9^/L. Cases with lymphocyte number = 0 were excluded from this study.

### 2.3 Observation indicators

1. To compare the Systemic Inflammatory Response Index (SIRI) levels between the favorable prognosis group and the non-favorable prognosis group.
2. To analyze the diagnostic performance of SIRI in differentiating severity levels of influenza A virus-induced pneumonia among elderly patients.

### 2.4 Statistical analysis

Statistical analysis was performed using SPSS software (version 26.0). Measured data were represented as mean value ± standard deviation (SD), and the inter-group comparisons were made with an independent samples *t*-test. Categorical data were represented as % and group comparisons were analyzed with the chi-square (χ^2^) test. Univariate analysis was performed using a logistic regression model, and the variables predictive value was assessed with receiver operating characteristic (ROC) curve analysis. The optimal cutoff value was calculated by identifying the cutoff point corresponding to the maximum Youden’s index (sensitivity + specificity − 1) across all cutoff points on the ROC curve. A *p*-value less than 0.05 was deemed statistically significant.

## 3 Results

### 3.1 Comparison of baseline characteristics

Retrospective analysis of 160 patients with influenza A virus-related pneumonia who were treated at the China Resources Wuhan Iron and Steel General Hospital between 24 December 2024 and 20 March 2025. Patients were divided into two groups based on their CURB-65 scores: favorable prognosis group (CURB-65 score <3) and non-favorable prognosis group (CURB-65 score ≥3). In total, 71 subjects were enrolled in the favorable prognosis group, and 89 subjects were included in the non-favorable prognosis group. Baseline clinical variables comparison between the two groups did not show a statistical difference (*p* > 0.05), which indicated that these two groups were homogeneous. For details, see Table 1.

**Table 1:** General information [*n* (%), *χ* ± s].

Basic information	Favorable prognosis group (*n* = 71)	Unfavorable prognosis group (*n* = 89)	*P*-value
Age, years	72.31 ± 6.989	77.29 ± 8.723	*P* < 0.001**
Height, cm	164.845 ± 8.146	168.219 ± 7.337	*P* = 0.007**
Weight, kg	63.38 ± 8.107	66.67 ± 8.118	*P* = 0.012**
WBC count, ×10^9^ /L	6.382 ± 3.022	8.351 ± 3.545	*P* < 0.001**
NEU count, ×10^9^ /L	4.402 ± 2.59	7.015 ± 3.363	*P* < 0.001**
LYM count, ×10^9^ /L	1.33 ± 0.567	0.759 ± 0.368	*P* < 0.001**
MONO count, ×10^9^ /L	0.487 ± 0.252	0.513 ± 0.221	0.492
HB, g/L	130.59 ± 18.146	125.35 ± 22.162	0.11
CRP, mg/L	25.638 ± 33.336	66.562 ± 70.821	*P* < 0.001**
PCT, ng/mL	0.347 ± 2.134	1.272 ± 5.44	0.145
IL-6, pg./mL	15.506 ± 22.504	176.327 ± 690.398	*P* = 0.031**
ESR, mm/h	22.031 ± 12.767	28.979 ± 22.727	*P* = 0.016**
PRE_Alb, g/L	179.66 ± 49.628	152.53 ± 57.552	*P* = 0.002**
Alb, g/L	40.701 ± 4.819	38.548 ± 4.866	*P* = 0.006**
TP, g/L	67.372 ± 5.822	64.349 ± 6.359	*P* = 0.002**
DBIL, μmol/L	3.985 ± 2.423	4.63 ± 2.87	0.132
TBIL, μmol/L	10.645 ± 5.648	11.3 ± 6.212	0.491
ALT, U/L	21.03 ± 13.882	22.3 ± 25.181	0.702
AST, U/L	25.124 ± 11.505	29.067 ± 24.967	0.188
BUN, mmol/L	5.939 ± 2.181	8.256 ± 3.72	*P* < 0.001**
Cr, μmol/L	74.752 ± 26.051	89.283 ± 40.475	*P* = 0.007**
UA, μmol/L	311.268 ± 96.433	312.958 ± 107.888	0.918
Hospital stay, days	5.54 ± 1.296	8.82 ± 3.325	*P* < 0.001**
SIRI	1.911 ± 1.784	6.007 ± 5.462	*P* < 0.001**
Sex	Male (37, 52.1%)	Male (67, 75.3%)	*P* = 0.002**
	Female (34, 47.9%)	Female (22, 24.7%)	
Smoking	Yes (29, 40.8%)	Yes (57, 64.0%)	*P* = 0.003**
	No (42, 59.2%)	No (32, 36.0%)	
HBP	Yes (29, 40.8%)	Yes (44, 49.4%)	0.278
	No (42, 59.2%)	No (45, 50.6%)	
TDM	Yes (10, 14.1%)	Yes (20, 22.5%)	0.177
	No (61, 85.9%)	No (69, 77.5%)	
CHD	Yes (14, 19.7%)	Yes (33, 37.1%)	*P* = 0.017**
	No (57, 80.3%)	No (56, 62.9%)	

** *p* < 0.05 was considered statistically significant. HBP, high blood pressure; TDM, diabetes; CHD, coronary heart disease.

### 3.2 Comparison of SIRI levels in the two groups of patients

The SIRI level in the group of elderly patients with influenza A virus-induced pneumonia and an unfavorable prognosis was significantly higher than in the group with a favorable prognosis (*p* < 0.05), as shown in Table 2.

**Table 2:** Comparison of SIRI levels between the two patient groups.

Group	Number of cases	SIRI (10^9^/L)
Favorable prognosis group	71	1.911 ± 1.784
Unfavorable prognosis group	89	6.007 ± 5.462
*p*	<0.001	

### 3.3 Analysis of SIRI’s discriminatory efficacy in assessing the severity of influenza A-induced viral pneumonia in the elderly population

Logistic regression model with classification of patients’ group as dependent variable (0 = the favorable prognosis group, 1 = the unfavorable prognosis group) and SIRI as independent variable was used. After adjustment for potential confounding factors, including sex, age, and smoking history, the association between SIRI and prognosis remained significant (*p* < 0.05), with a 95% confidence interval (CI) for the odds ratio (OR) that did not cross 1. Specifically, each unit increase in SIRI was associated with an 80% increase in the risk of an unfavorable prognosis (OR = 1.800, 95% CI: 1.254–2.828). These results suggest that SIRI is a statistically significant independent predictor of disease severity in elderly patients with influenza A-associated viral pneumonia, as shown in Table 3.

**Table 3:** Evaluation of SIRI in assessing the severity of influenza A-induced viral pneumonia among elderly patients, along with the results of the discriminative performance analysis.

Variable	*P*	OR	95%CI
SIRI	0.004	1.800	1.254 ~ 2.828
Sex, female	0.892	0.890	0.165 ~ 5.093
Age, years	0.425	1.033	0.953 ~ 1.121
BMI, kg/m^2^	0.173	1.215	0.925 ~ 1.639
Smoking, yes	0.838	0.852	0.178 ~ 4.043
HBP, yes	0.656	0.749	0.205 ~ 2.669
TDM, yes	0.303	2.234	0.497 ~ 10.925
CHD, yes	0.161	2.694	0.688 ~ 11.301
SBP, mmHg	0.927	1.002	0.960 ~ 1.044
DBP, mmHg	<0.001	0.889	0.830 ~ 0.942
WBC count, ×10^9^ /L	0.368	0.879	0.647 ~ 1.146
HB, g/L	0.254	1.020	0.986 ~ 1.057
PCT, ng/mL	0.351	1.131	0.925 ~ 1.463
IL-6, pg./mL	0.505	1.003	0.999 ~ 1.017
ESR, mm/h	0.375	1.015	0.983 ~ 1.052
BNP, pg./mL	0.417	1.001	0.999 ~ 1.003
ALT, U/L	0.626	1.018	0.949 ~ 1.092
AST, U/L	0.763	1.010	0.946 ~ 1.082
Cr, umol/L	0.081	0.975	0.947 ~ 1.003
BUN, mmol/L	0.002	1.928	1.317 ~ 3.017
UA, umol/L	0.068	0.993	0.985 ~ 1.000
PRE_Alb, g/L	0.855	1.001	0.987 ~ 1.016

**Figure 1:**
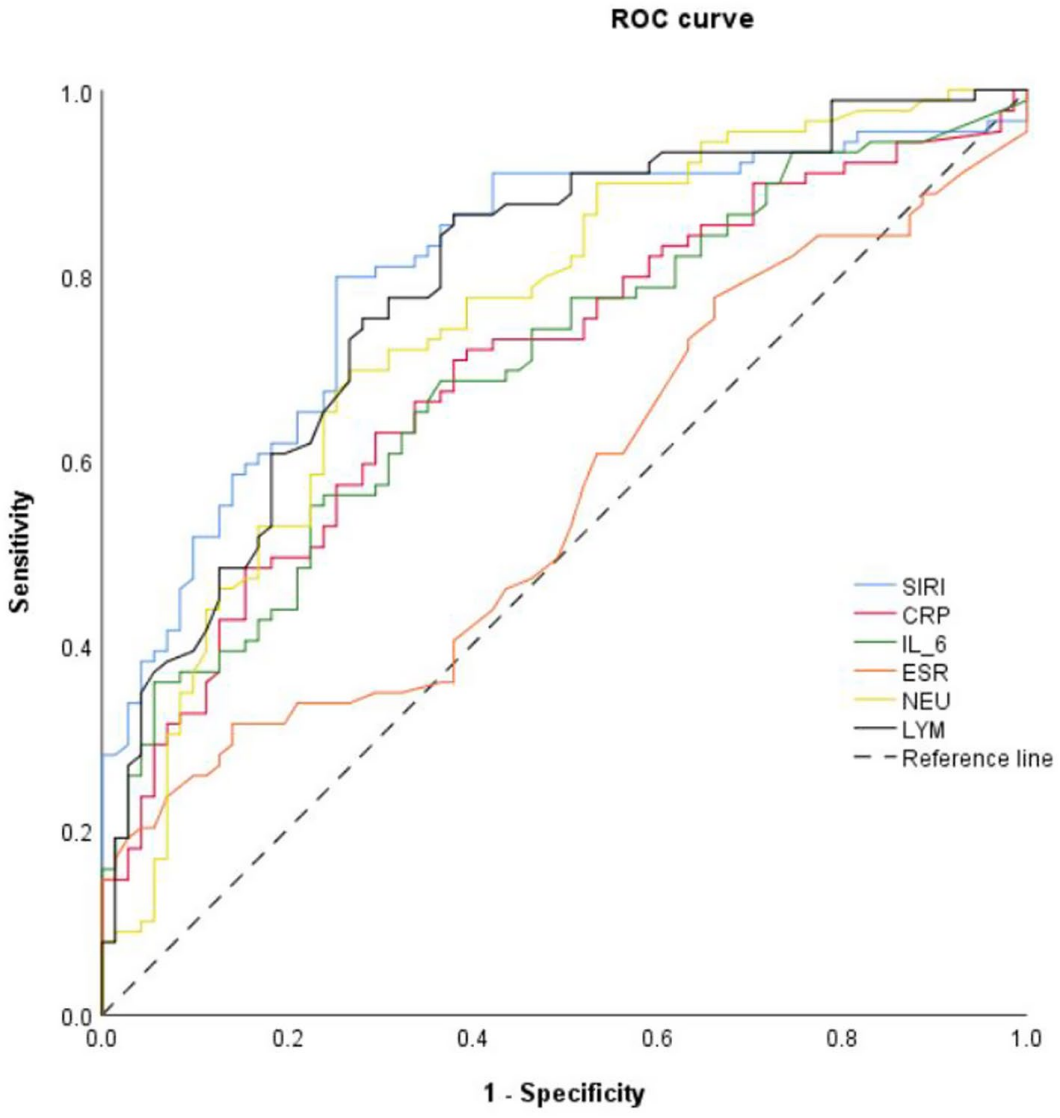
ROC curve of SIRI for predicting the severity of influenza A virus-induced viral pneumonia in elderly patients.

SIRI levels were utilized to forecast the course of the patients’ condition progression to severe disease by means of ROC curve analysis, and compared with the predictive performance of CRP, IL-6, ESR, neutrophil (NEU) counts, and lymphocyte (LYM) counts, which shows that SIRI had the largest area under ROC curve (AUC = 0.806) as well as the largest Youden index (J = 0.544). The results prove that SIRI possesses good discrimination ability to differentiate the severity of influenza A virus-induced pneumonia in the elderly, as shown in Table 4 and Figure 1.

**Table 4:** Severity assessment of influenza A virus-induced viral pneumonia in elderly patients: prediction of associated ROC curve parameters.

Variable	Optimal cutoff value	Area under the ROC curve (AUC)	Diagnostic sensitivity/%	Diagnostic specificity/%	Youden Index/J
SIRI	0.448	0.806	0.798	0.746	0.544
CRP, mg/L	0.500	0.701	0.629	0.704	0.333
IL-6, pg./m L	0.508	0.701	0.551	0.775	0.326
ESR, mm/h	0.608	0.561	0.315	0.859	0.174
NEU, ×10^9^ /L	0.530	0.752	0.674	0.746	0.421
LYM, ×10^9^ /L	0.490	0.790	0.865	0.620	0.485

## 4 Discussion

Accumulating evidence indicates that the pathogenesis of influenza A virus pneumonia (IAVP) is regulated by a “double-edged sword” mechanism involving direct viral damage and the host immune-inflammatory response. While the inflammatory response is essential for viral clearance, an excessive reaction may lead to extensive tissue damage and multiple organ failure (11). Thus, the immune-inflammatory response is a crucial determinant of clinical outcomes in IAVP.

As a novel composite inflammatory biomarker, the Systemic Inflammatory Response Index (SIRI) integrates key cellular components of both innate and adaptive immunity, thereby overcoming the limitations of single-parameter markers. Derived from neutrophil, monocyte, and lymphocyte counts, SIRI captures three critical steps of the host immune response: immediate pathogen defense (neutrophils), antigen presentation (monocytes), and specific adaptive immunity (lymphocytes). Elevated neutrophil counts typically reflect acute tissue damage and pro-inflammatory mediator release; monocytosis is associated with tissue infiltration and prolonged inflammation; and lymphopenia often indicates immune exhaustion or suppression. This multi-dimensional structure enables SIRI to comprehensively characterize systemic inflammation (12). Notably, in elderly patients, age-related thymic atrophy leads to decreased lymphocyte counts, while monocytes and neutrophils tend to increase. These physiological changes may increase variability in SIRI values and support its clinical utility in older populations (13).

In this study, SIRI levels were significantly higher in the unfavorable prognosis group than in the favorable prognosis group (*p* < 0.05), and SIRI was identified as an independent predictor of disease severity after adjustment for potential confounders. These findings suggest that SIRI is closely associated with disease progression in elderly patients with IAVP, with elevated levels potentially reflecting an exaggerated inflammatory state, secondary infection, or multi-organ dysfunction.

Several limitations of this study should be acknowledged. First, as with all composite indices, SIRI does not capture the functional and maturational heterogeneity of leukocyte subsets. Neutrophils, in particular, exhibit substantial heterogeneity during severe inflammation, with distinct subpopulations (e.g., immature granulocytes and low-density neutrophils) that may play either pro-inflammatory or immunosuppressive roles (14). Although Cancella De Abreu et al. (15) evaluated advanced “cell population data” parameters to capture this heterogeneity for early sepsis detection, these parameters did not demonstrate sufficient diagnostic accuracy for routine screening. In the present retrospective analysis, we were unable to account for such cellular heterogeneity. Therefore, while SIRI is a practical and independently predictive marker, it should be interpreted as a summary measure of global inflammation-immunity balance rather than as a reflection of specific leukocyte subset dynamics. Second, this study did not measure serum levels of interleukin-10 (IL-10) or transforming growth factor-beta (TGF-*β*). Both cytokines have been reported to play important roles in the immunopathology of severe viral infections, including influenza, where an imbalance between pro-inflammatory and regulatory responses may influence clinical outcomes (16). The absence of these cytokine data was primarily due to the constraints of routine clinical practice and the relatively high cost of these assays, which precluded their inclusion in standard admission workups. Third, this study used a cross-sectional design, precluding the evaluation of dynamic changes in SIRI with respect to disease progression. Fourth, the single-center, retrospective design and relatively small sample size limit the generalizability of our findings, and serial measurements of SIRI over time were not available.

Binary logistic regression analysis confirmed that SIRI is an independent predictor of disease severity in elderly patients with IAVP. ROC curve analysis demonstrated that SIRI possesses good predictive performance, with an area under the curve of 0.806, outperforming traditional inflammatory markers such as CRP, IL-6, ESR, neutrophil count, and lymphocyte count. Given these inherent limitations, however, the use of SIRI in combination with other predictive markers is recommended in clinical practice to improve diagnostic precision.

Future prospective studies incorporating flow cytometry or single-cell technologies are warranted to further elucidate the roles of neutrophil and monocyte subsets in this vulnerable population. In addition, larger, multi-center, prospective studies are required to validate and generalize our findings and to investigate the relationship between SIRI, key immunomodulatory cytokines (including IL-10 and TGF-*β*), and disease severity in elderly patients with IAVP.

In conclusion, SIRI may serve as a valuable, practical predictive marker of disease severity in elderly patients with influenza A virus-induced pneumonia. Early assessment of SIRI could facilitate the identification of patients at high risk of severe illness, guide timely antiviral and anti-inflammatory therapy, and ultimately contribute to improved clinical management and prognostication. Notably, SIRI offers distinct advantages for primary healthcare settings: it is cost-effective, derived from routine complete blood counts, and simple to calculate. Several limitations should be acknowledged, including the retrospective single-center design, the lack of longitudinal data, and the absence of IL-10 and TGF-*β* measurements. Despite these limitations, SIRI remains a reliable and objective biomarker for assessing disease severity in this vulnerable population.

## Data Availability

The original contributions presented in the study are included in the article; further inquiries can be directed to the corresponding author.
